# Predictive context biases binocular rivalry in children and adults with no positive relation to two measures of social cognition

**DOI:** 10.1038/s41598-020-58921-8

**Published:** 2020-02-06

**Authors:** Christian Valuch, Louisa Kulke

**Affiliations:** 1 Department of Experimental Psychology, University of Goettingen, Goettingen, Germany; 2 Department of Affective Neuroscience and Psychophysiology, University of Goettingen, Goettingen, Germany; 3 Leibniz ScienceCampus Primate Cognition, Goettingen, Germany

**Keywords:** Consciousness, Human behaviour

## Abstract

Integration of prior experience and contextual information can help to resolve perceptually ambiguous situations and might support the ability to understand other peoples’ thoughts and intentions, called Theory of Mind. We studied whether the readiness to incorporate contextual information for resolving binocular rivalry is positively associated with Theory-of-Mind-related social cognitive abilities. In children (12 to 13 years) and adults (18 to 25 years), a predictive temporal context reliably modulated the onset of binocular rivalry to a similar degree. In contrast, adult participants scored better on measures of Theory of Mind compared to children. We observed considerable interindividual differences regarding the influence of a predictive context on binocular rivalry, which were associated with differences in sensory eye dominance. The absence of a positive association between predictive effects on perception and Theory of Mind performance suggests that predictive effects on binocular rivalry and higher-level Theory-of-Mind-related abilities stem from different neurocognitive mechanisms. We conclude that the influence of predictive contextual information on basic visual processes is fully developed at an earlier age, whereas social cognitive skills continue to evolve from adolescence to adulthood.

## Introduction

Imagine sitting on a train that has just stopped at a station. You gaze out of the window and see the carriages of another train at the adjacent track. After a while, you feel that your train is leaving the station, only to realize moments later that it is the other train moving while yours is still standing. In this case, you incorrectly attributed your visual sensation to a plausible external cause. However, the likelihood of your interpretation was high, given that the window view changes are similar, irrespective of which of the two trains move. Misinterpretations of the causes of sensory experiences do not happen very frequently in everyday life, because the world around us changes in somewhat predictable ways and ambiguous situations such as in the example above are rare. Our knowledge and prior experience help us to perceive and understand what happens around us so that we can act and respond appropriately and adaptively. The idea that prior experience shapes perception can be traced back to the seminal work on human perception by von Helmholtz^1^. More recently, it became the cornerstone of predictive coding models of perception and cognition^2–4^. Predictive coding models assume cortical processing hierarchies in which later processing stages generate predictions that are communicated back to earlier processing stages, where they are compared against the incoming signal from yet earlier processing stages. At each level, the mismatch between predictions and the current representation of sensory signals is communicated to later stages where it updates the predictions. By minimizing the prediction error across all processing stages, the brain establishes a stable state, which could be the basis for recognizing intricate sensory patterns^5,6^ and conscious perceptual experience in general^7–9^.

To study factors involved in conscious perceptual experience under laboratory settings, researchers have often employed binocular rivalry (BR)^1,8,10,11^. BR is a fundamental perceptual phenomenon that occurs under dichoptic viewing conditions when the left- and the right eye of the same individual are confronted with different visual stimuli. The conflict between the incoming sensory signals entails a dynamically changing perceptual experience in which viewers do not see both stimuli at the same time. Instead, one of the rivalrous stimuli is perceptually dominant, while the other stimulus is invisible. After some time, however, a perceptual switch occurs, and the second stimulus becomes dominant while the first one becomes invisible. In between these phases of exclusive dominance, participants usually experience phases of mixed dominance, in which parts of both stimuli can be visible^12^. Despite establishing the same stimulus conditions, perceptual dynamics vary considerably between individual participants^13,14^. For example, perception during BR might undergo specific developmental changes from childhood to adulthood^15–17^. Hudak *et al*.^16^ compared rivalry dynamics in 9-year old, and 12-year-old children with a group of 21-year-old adults. Their data yielded a developmental trend in dominance times, with longer dominant percept durations and slower perceptual switch rates in adults compared to children. Moreover, rivalry time courses were characterized by a stronger influence of the cumulative perceptual history in children compared to adults^16^. Also, specific differences in rivalry dynamics were found for children on the autism spectrum compared to typical children^18–20^. Related to these findings, a recent study reported that at around 12 years of age, neurotypical children integrate social contextual information to make accurate perceptual decisions about ambiguous stimuli, while no comparable developmental step could be observed in children on the autism spectrum^21^. These studies suggest that basic perceptual processes undergo developmental changes from childhood to adulthood, and individual differences in the readiness to integrate contextual information into perceptual decisions might be associated with social cognitive abilities.

To experimentally test the integration of contextual information into perception, Denison *et al*.^22^ used a novel type of BR paradigm. Their participants saw a sequence of simple, oriented grating stimuli, which rotated, in several discrete steps, either clockwise or counterclockwise. This predictive rotation sequence was followed by two differently oriented gratings, presented to the left and the right eye separately, establishing the conditions for BR. Critically, one of the rivalrous gratings matched the predictive context in the sense that it was the next step in the rotation sequence, whereas the other grating had the orthogonal orientation. In the majority of trials, the participants’ first exclusive percept at the onset of BR was the grating that matched the predictive context, whereas the orthogonal grating was perceived only in the minority of trials^22^. This finding can be interpreted as psychophysical evidence for predictive coding at an early perceptual processing stage^8,22–24^. Related to this result, other studies reported that the initial percept at the onset of BR could also be modulated by statistical regularities of learned stimulus associations^24,25^. Reliable contextual effects at the onset of BR are particularly interesting because this early stage of BR has been previously considered somewhat immune to top-down modulations^26^. However, so far, it is not known whether these perceptual predictive coding effects also undergo developmental changes from childhood to adulthood, in line with more general age differences in BR dynamics^15–17,21^. Moreover, given the hypothesized links between basic perceptual processing and autism^18,21^, it would be informative to assess whether individual variability in predictive context effects are linked to individual variability in social cognitive skills.

Reliable integration of prior experience and contextual information through predictive coding might also be critical for higher perceptual and social cognitive skills associated with Theory of Mind (ToM)^27,28^. ToM is defined as the ability to recognize the beliefs, desires, or internal states of other persons. Similar to the perception of ambiguous stimuli^16,21^, ToM has a typical developmental trajectory. Explicit ToM develops around the age of 4 years, when children pass simple tasks in which they need to infer an agent’s mental state from a story^29,30^ but continues to improve until adulthood^31^. Even in adulthood, ToM skills vary between individuals^32^, with particular deficits in people with autism^33,34^. Inferring others’ mental states and intentions might require internal models of other individuals, social situations as well as the integration of contextual information. Higher-level cortical networks for the inference of others’ beliefs and intentions can include the mirror neuron system^27^ and the temporoparietal junction^35^, which can be integrated into a predictive coding framework. Assuming predictive coding as a general cortical processing principle, higher levels of processing could interact with perceptual processes at early visual processing stages^36^. Accordingly, a relationship between predictive coding at earlier and higher stages of the processing hierarchy could provide a model for understanding specific social deficits of people with autism^21,37–39^.

The aims of our study were two-fold: first, we wanted to test if predictive context effects on BR^22^ undergo developmental changes from childhood to adulthood. Second, we wanted to assess if individual differences in predictive context effects are associated with ToM abilities. A positive relation between predictive context effects in perception and ToM would suggest that an individual disposition to incorporate contextual information is important for more complex perceptual and social judgments. Moreover, our study explored the use of BR for assessing developmental changes in a potentially critical ToM-related perceptual mechanism, which could serve as a potential implicit measure of ToM^40^. The current study used two established test instruments for assessing individual variability in ToM. The Reading Mind in the Eyes (RME) test^41^ uses greyscale pictures and is, therefore, more related to specific perceptual processes. In contrast, the Strange Stories test (SST)^33^ uses short narrations and, therefore, measures higher cognitive and reasoning skills related to ToM. If BR onset effects and performance in ToM reflected a common individual disposition to incorporate prior experience into perceptual judgments, we expected a positive association between predictive context effects in perception and ToM measures. In our analyses, we first assessed whether perceptual dynamics and predictive context effects in BR undergo developmental changes from childhood to adulthood. Second, we assessed whether ToM skills improve with age and whether individual differences converge across different ToM scales. Finally, we analyzed whether predictive context effects on BR were positively associated with individual ToM performance. In this joint analysis, we also controlled for effects of sensory eye dominance, as these represent an independent source of individual variability for BR, in addition to the integration of a predictive context^13^.

## Methods

### Participants

A total of N = 91 participants were recruited from two age populations. For the group of adults, N = 55 (35 female, 20 male) participants between 18 and 25 years (M = 22.1 years) were recruited via posters, leaflets, and social networking sites. Adults volunteered to participate in return for course credit or monetary compensation (8 €) after written informed consent was given. For the group of children, N = 36 participants (21 female, 15 male) between 12 and 13 years (M = 153.8 months) were recruited through the children participant database of the developmental psychology department. Parents signed an informed consent form. Children volunteered to participate after signing an informed assent form and received a small age-appropriate toy as a thank you gift. Two participants (1 adult, 1 child) were excluded from the data analyses because they did not report any clear percepts during the 30-s binocular rivalry trials. All participants had normal or fully corrected visual acuity. For each participant, the overall test session took approximately 1 hour. All study protocols adhered to the tenets of the Declaration of Helsinki and were in accordance with the ethical standards of the German Psychological Society. All study protocols were approved by the ethics committee of the Georg-Elias-Mueller-Institute for Psychology of the University of Goettingen (reference no.: 181).

### Apparatus

BR experiments were conducted using a FOVE (Fove Inc., San Mateo, CA) head-mounted-display (HMD). The HMD was fit to the participant’s head using adjustable velcro straps. Visual stimuli were rendered at the display’s native resolution and frame rate of 2, 560 × 1, 440 pixels at 70 Hz. For dichoptic stimulation, the rivalrous stimuli were presented at different locations side-by-side on the HMD screen while each stimulus could only be viewed by one eye through the left and right HMD lenses. For non-dichoptic stimulation (during the presentation of the predictive context stimulus sequence or during catch trials), the same stimulus was presented at both screen locations. Experimental procedures were programmed in PsychoPy2 version 1.85.2^42^, and run on an Intel-based PC system under Microsoft Windows. Manual perceptual reports and discriminations were collected using a USB-connected numerical keypad. Participants used a left button (key ‘4’) to report exclusive perception of left-oriented stimuli, a right button (key ‘6’) to report perception of right-oriented stimuli and a down button (key ‘2’) to start the next trial. During the experiments, participants held the keypad with both hands and responded with the left- and the right thumb, respectively. During perceptual reports, key states were continuously polled at a rate of 200 Hz.

### Binocular rivalry experiments

#### Visual stimuli

Two screen areas at which experimental stimuli were presented encompassed 8.4 × 8. 4° and had a 50% neutral gray background. All pixels outside these areas remained black during the entire experiment. To facilitate stable vergence, we included a slightly darker (40% neutral gray) circular annulus with a diameter of 7. 5° and a line strength of 0. 4°. The center of the annuli contained a small fixation dot of 0.15° of the same gray value as the annulus. As experimental stimuli, we used sinusoidal luminance gratings presented in a circular Gaussian window. The gratings had a spatial frequency of 1 cycle/° and a spatial extent of 5.75 × 5.75°. This combination of spatial frequency and spatial extent of the grating stimuli should produce reliable exclusive dominance epochs^43^, which was confirmed in informal piloting runs before starting data collection. The Michelson contrast of all grating stimuli was set to 10%. The gratings were oriented vertically (0°), horizontally (90°), or diagonally (±45° of the vertical axis). During phases of perceptual reports, only diagonally oriented gratings were shown, and the participants reported whether they perceived a left-oriented (−45°) or right-oriented (+45°) grating.

#### Consecutive rivalry experiment

**Figure 1:**
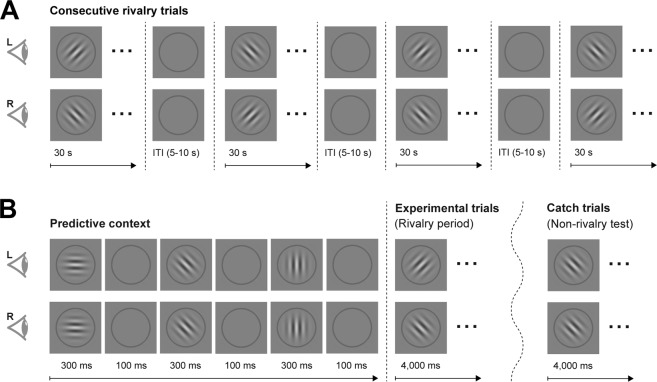
Schematic of the trial procedure during the binocular rivalry experiments. (**A**) In the consecutive rivalry experiment, participants completed four 30s-trials with different gratings presented to the left eye (L) and the right eye (R), respectively. Participants continuously reported whether their dominant percept was a right-oriented grating, or a left-oriented grating. Inter-trial intervals lasted about 5 to 10 s, depending on when the participants decided to start the subsequent trial. Between trials, the stimulus-to-eye mapping switched. (**B**) In the predictive context experiment, participants completed 96 experimental trials, which always started with the presentation of a sequence of three non-rivalrous grating stimuli separated by an inter-stimulus interval. The predictive context was followed by a 4-s rivalry period in which different orthogonal gratings were presented to the left and the right eye. Participants reported the dominant percept. If the predictive context influenced BR, the stimulus that matched the predictive sequence (here, the grating presented to the left eye) should be perceived in the majority of trials. Participants also completed 24 non-rivalrous catch trials (randomly intermixed with experimental trials) which were identical to the experimental trials except that the same stimulus was shown to both eyes during the perceptual report period. Catch trials were included to verify that participants performed the task correctly.

The first experiment consisted of the consecutive presentation of static rivalrous stimuli for 30 s per trial (see Fig. 1A). This experiment was conducted for two reasons. First, we wanted to assess general characteristics of BR (i.e., number of perceptual switches, exclusive percept epoch durations, and durations of no reported exclusive percepts) and test for potential differences between age groups, which were sometimes reported in previous research. Second, we wanted to determine the sensory eye dominance for individual participants, depending on the total perceptual dominance durations of stimuli perceived with the left eye vs. the right eye which is a robust indicator of eye dominance in BR^44^. The participants’ task was to continuously report, using the keypad buttons, whether they currently experienced the left- or the right-oriented grating as perceptually dominant. Participants were instructed not to press any buttons during phases of mixed or unclear percepts. At the end of the rivalry period, the gratings disappeared, and a dark gray fixation dot was shown for an interval of one second. Following this minimum inter-trial interval, the fixation dot changed its color to green, indicating to the participants that they could start the next trial using the down button, as soon as they felt ready. Before starting the experiment, participants were carefully explained the task and the stimulus-to-response mapping using three demonstration trials, one with non-rivalrous left-oriented gratings, one with non-rivalrous right-oriented gratings and one with rivalrous orthogonal gratings.

#### Predictive context experiment

For the second and main experiment, we developed a child-friendly version of the procedure of Denison *et al*.^22^. The purpose of this experiment was to measure the extent to which BR onset would be influenced by a predictive context (see Fig. 1B). Participants started each trial by pressing the ‘down’ button on the keypad. After a blank period of 0.2 s, the trial started with a predictive context, consisting of a sequence of three static gratings presented for 300 ms each, which were separated by a 100 ms inter-stimulus-interval during which no gratings were shown. The gratings generated the impression of a clockwise- or counterclockwise-rotating sequence. The first grating of the predictive sequence was always horizontal, followed by a grating that was tilted 45° clockwise in relation to the horizontal (for the right-rotating sequence, see the example in Fig. 1A) or 45° counter-clockwise (for the left-rotating sequence). The third grating of the predictive context was always vertical. Following another blank of 100 ms, two rivalrous gratings (±45° from the vertical) were presented dichoptically for 4 s, and the participants’ task was to report the perceptually dominant grating during this rivalry period. Each trial ended after the 4-s reporting period. After each trial, a green fixation dot was presented, indicating to the participants that they could start the next trial whenever they felt ready.

The experiment tested the hypothesis that the first dominant percept would be influenced by the predictive rotation sequence^22^. The experiment consisted of 96 experimental trials (with rivalrous gratings) and 24 catch trials (with non-rivalrous gratings) during the reporting period. Catch trials were included to verify that participants understood the task instructions and did not merely report the rotation direction of the predictive context (in that case, the participant’s performance would be at chance level in the catch trials because only in half of the catch trials the test gratings continued the predictive context sequence). Experimental and catch trials were presented randomly intermixed across the length of the experiment, and all combinations of rotation sequences and stimulus-eye mappings were presented with an equal frequency. The order of trials and conditions was fully randomized for each participant. Before starting with this experiment, participants were shown 16 demonstration trials and carefully explained the task.

### Theory of mind measures

Two often-used tests measuring different perceptual and cognitive aspects of ToM-related skills were used. The Reading the Mind in the Eyes test (RME)^41^ presents participants with 36 images depicting the eye-region of a face. Participants need to choose one out of four words describing the mental state that this face expresses. A German pen-and-paper version of this test was administered, and the number of correctly identified expressions was computed as a performance score. As the RME presents participants with greyscale pictures of face parts, it relates to specific perceptual processes involved in ToM. In addition, the Strange Stories test (SST)^33^ was conducted as a more abstract cognitive test of ToM. In this test, participants read out stories describing situations in which the protagonist’s behavior can either be explained based on mental states, or physical events. They are then asked to justify the protagonist’s behavior either based on undescribed mental states (ToM scale) or causal physical relations (physical scale). Based on previous research^45^, a selection of nine stories adapted from the original SST^32,33^ and a modified German version^46^, controlled for number of words and semantic and syntactic complexity, were presented to participants. Participants’ justifications were scored according to previous research, as either entirely correct (2 points), partially correct (1 point) or incorrect (0 points)^33,45^. Participants’ answers during the SST were rated by one coder during the test session and audio-recorded and scored offline by a second rater. Both raters agreed on the SST ToM and SST Physical scores in 85% of all participants (89.7% within adults, and 77.8% within children). If raters did not agree, an average score of their judgments was assigned to that participant.

### Data analysis

**Figure 2:**
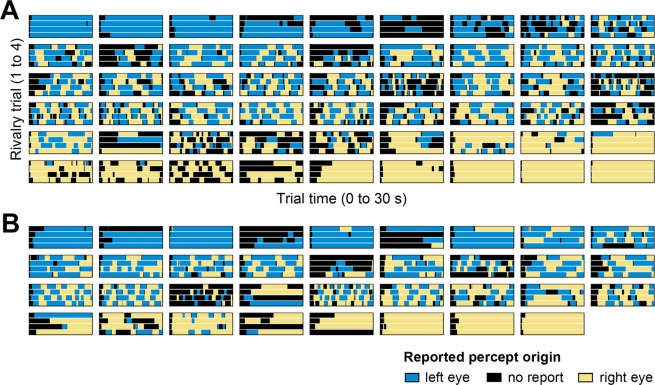
Perceptual dynamics during the consecutive rivalry experiment in adults (**A**) and children (**B**). Participants completed four rivalry trials of 30 s each. Each subplot depicts the continuous perceptual report from one individual participant during each of the four trials (ordinates) across the duration of the trial (abscissae). Colors demark epochs in which the stimulus presented to the left eye was reported as dominant (blue), epochs in which the stimulus presented to right eye was reported as dominant (yellow), and epochs during which none of the stimuli was reported as exclusively dominant (black). Within each group, participants were ordered according to their sensory eye dominance from strongly left eye dominant participants to strongly right eye dominant participants.

Continuous perceptual reports in the BR experiments were parsed into phases of exclusive perceptual dominance (periods during which only one of the response buttons was pressed), and phases of no reported exclusive dominance (periods during which both response buttons or no response button was pressed). At the individual participant level, we computed the median exclusive dominance epoch duration and the median ‘no report’ epoch duration based on all epochs collected from the four trials. We also computed the average number of perceptual switches and the overall ‘no report’ duration per trial. Perceptual switches were defined as transitions from no reported exclusive dominance to an exclusive percept or direct transitions from one exclusive percept to the other exclusive percept. To assess individual eye dominance, we computed the total exclusive percept duration of stimuli presented to the right eye and compared it to the total exclusive percept duration of stimuli presented to the left eye. As the dominant eye, we defined the eye with an overall higher total dominance duration. Individual participant data from the continuous rivalry experiment is plotted in Fig. 2 (ranked from left-eye-dominant participants to right-eye-dominant participants).

In line with previous research, we assessed the effect of predictive context on BR by analyzing the proportion of trials in which the percept that matched the predictive context was reported as the initial exclusive percept at the onset of BR^22–25^. Trials in which participants did not report any clear percepts were excluded from the analyses (i.e., 16.1% of experimental trials in adults, 13.1% in children). The percentage of trials in which the initial percept matched the predictive context was computed per participant. Across participants, we tested whether this percentage was significantly higher than 50%. We also tested whether the matching effect differed between age groups and depending on whether the matching grating was presented to the dominant or the nondominant eye of the participants. For testing between-group differences, we computed Welch’s t-tests with approximated degrees of freedom based on the Welch-Satterthwaite equation. To test for associations between predictive context effects on BR and ToM performance while simultaneously controlling for individual differences in eye dominance, we analyzed the binary outcome variable of the initial percept (1 = matching predictive context vs. 0 = not matching predictive context) at the single-trial level using a generalized linear mixed model (GLMM) approach, as implemented in the R package ‘lme4’^47^. The GLMM included continuous predictor variables for individual differences in ToM and eye imbalance, which were Z-scaled in order to model the graded individual differences within age groups. In general, we assumed P values below an *α* of 0.05 as statistically significant (all tests were two-tailed). All analyses were performed using R 3.6.1 (https://www.R-project.org/).

## Results

### Perception during binocular rivalry

#### Consecutive rivalry and eye dominance

Individual perceptual dynamics across the duration of the four consecutive rivalry trials are plotted in Fig. 2A (for adults) and 2B (for children). Individual data showed considerable variability in sensory eye dominance in both groups of participants. Both age groups included participants with a pronounced imbalance of one eye over the other (i.e., predominant perception of stimuli presented to the left, or to the right eye, respectively), and a larger number of participants with more balanced eye dominance, which showed more frequent perceptual switches between the left- and the right eye’s input over the course of the individual trials. Table 1 summarizes general characteristics of the BR dynamics in both age groups. The statistical analyses revealed no differences between age groups with regard to the average number of perceptual switches per trial, the median exclusive dominance duration, or the duration of epochs in which no exclusive percept was reported.

**Table 1:** General characteristics of perceptual dynamics based on the data from the consecutive rivalry experiment in adults and children. Group values represent means and standard errors across participants.

Measure at the participant level	Group values		Difference (Welch’s t-test)		
	Adults	Children	t	df	P
Median exclusive percept epoch duration (s)	7.66 (1.12)	10.51 (1.73)	−1.39	61.7	0.171
Median ‘no report’ epoch duration (s)	1.57 (0.30)	2.44 (0.73)	−1.10	45.5	0.277
Number of switches per trial	3.75 (0.36)	3.18 (0.51)	0.90	65	0.373
Overall ‘no report’ duration per trial (s)	8.08 (0.79)	7.32 (1.1)	0.56	66.8	0.575

#### Predictive context modulates binocular rivalry

**Figure 3:**
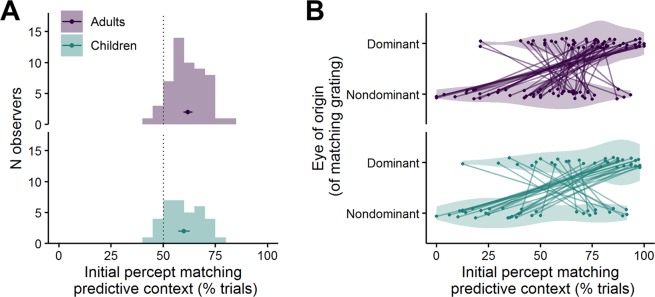
Predictive context biases the onset of binocular rivalry in adults and children to a similar degree. (**A**) Overall percentage of trials in which the initial percept matched the predictive context, averaged across all trials. The single data point in each subplot (top: adults, bottom: children) represents the mean and 95% confidence interval of the respective group. (**B**) Predictive context effects, split for trials in which the matching grating was presented to the dominant vs. the nondominant eye. Data points belonging to the same participants are connected with lines to illustrate that eye dominance also plays a role for the overall matching effect.

Across both age groups, the stimulus matching the predictive context was reported as the initial dominant percept in 61.1% of all trials, suggesting a significant influence of the predictive context, t(88) = 12.9, P < 0.001 (t-test against 50%). As illustrated in Fig. 3A, the effect was significant both within adults, M = 61.8%, t(53) = 10.9, P < 0.001 as well as within children, M = 59.9%, t(34) = 7.0, P < 0.001, and the overall effect did not differ between the two age groups, t(69.9) = 1.1, P = 0.296.

Due to the large inter-individual variability in sensory eye dominance in our sample of participants (Fig. 2) and the fact that eye dominance is a known determinant of BR dynamics^13,44,48,49^, we conducted a follow-up analysis in which we considered whether the grating matching the predictive context was presented to the nondominant or the dominant eye, as an additional within-participant variable. Figure 3B illustrates that the matching effect was strongly modulated by eye dominance but the strength of this modulation varied between individual participants. A mixed ANOVA confirmed that across groups, the eye of origin of the matching grating had a significant effect on the matching effect, F(1, 87) = 34.1, P < 0.001 (with a matching effect of 73.6% for the dominant eye vs. 46.1% for the nondominant eye). There was no interaction of Eye of Origin × Group, F(1, 87) = 0.21, P = 0.648, suggesting that eye of origin played a similar moderating role in both age groups.

Due to the balanced experimental design, the asymmetry between the nondominant and the dominant eye does not limit the overall reliability of the matching effect at the group level. However, because we want to relate individual BR onset effects to individual ToM scores, we consider it necessary to control for eye dominance effects as an additional source of individual differences. This is relevant for two reasons. First, low matching effects (across both eyes) could result from strong eye dominance, because participants with pronounced eye dominance might report the stimulus that is presented to the dominant eye in most trials, whether it matches the predictive context or not. Hence, part of the variance in individual matching effects could be explained by sensory eye dominance. Second, participants did not always provide a perceptual report, resulting in partially unbalanced datasets at the participant level (unequal numbers of perceptual reports from trials in which the matching grating was presented to the dominant vs. the nondominant eye). Hence, the individual matching effects could be, at least for some participants, confounded with sensory eye dominance effects. Thus, the matching effect across trials might not be a pure estimate for an individual’s tendency to incorporate the predictive context into perception, which is why we controlled for eye dominance when evaluating the relationship between BR onset effects and ToM performance.

#### Performance in catch trials

Performance in catch trials was close to perfect in adults (correct responses M = 99.2%, SE = 0.3) as well as in children (M = 98.1%, SE = 0.59) with no significant difference in performance between the two observer groups, t(51.5) = 1.63, P = 0.109. The test grating was reported significantly better than chance in both the group of adults, t(53) = 163.9, P < 0.001 as well as in the group of children, t(34) = 81.2, P < 0.001. This suggests that both groups performed the task as instructed, and neither of the groups merely reported the rotation direction of the predictive context.

### Performance in theory of mind tasks

We first tested whether performance on ToM scales improved with age. A Welch t-test showed that adults (M = 25.7, SE = 0.46) significantly outperformed children (M = 22.0, SE = 0.8) on the RME test as a perceptual ToM measure, t(56.1) = 4.1, P < 0.001. There also was a tendency for adults to perform better on the ToM score of the SST (adults M = 5.3, SE = 0.20; children M = 4.8, SE = 0.20), t(81.6) = 1.9, P = 0.066. There was no difference between age groups on the physical items of the SST (adults M = 6.3, SE = 0.19; children M = 5.8, SE = 0.28), t(62.2) = 1.3, P = 0.193.

**Figure 4:**
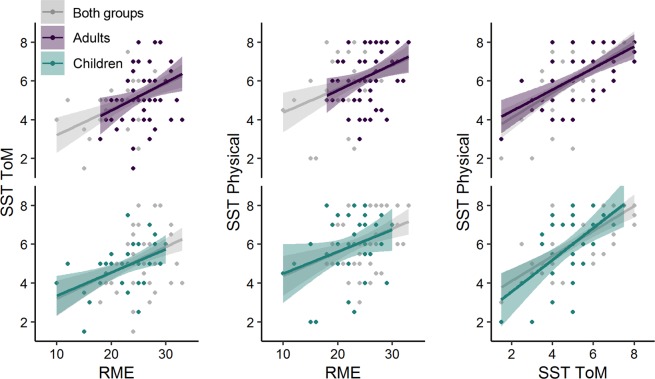
Correlations between the different ToM measures, across and within groups. Upper panels (in purple): Correlations within the group of adults. Lower panels (in green): Correlations within the group of children. Both panels (in gray): Correlation across both participant groups. For coefficients, see main text. RME, Reading the Mind in the Eyes. SST ToM, Strange Stories Test - Theory of Mind.

Correlation analyses (see Fig. 4) showed significant correlations of the RME with the SST ToM scale, r(87) = 0.42, P < 0.001 and with the SST physical scale, r(87) = 0.35, P < 0.001. The sub-scales SST ToM and SST physical also correlated significantly, r(87) = 0.59, P < 0.001. Separate correlation analyses for both age groups showed similar results. In children there were significant correlations of the RME with the SST ToM scale, r(33) = 0.47, P = 0.004 and between the sub-scales SST ToM and SST physical, r(33) = 0.59, P < 0.001. The correlation between the RME and SST physical score within children was not statistically significant, r(33) = 0.32, P = 0.060. In adults, there were significant correlations of the RME with the SST ToM scale, r(52) = 0.34 P = 0.012, and with the SST physical scale, r(52) = 0.33, P = 0.014. The sub-scales of the SST also correlated significantly in adults, r(52) = 0.59, P < 0.001.

### Perception and theory of mind

To assess the relationship between predictive context effects on basic perceptual processing and higher perceptual skills related to social understanding (as measured by ToM scales), we analyzed the different measures collected from each participant in a joint statistical model. To control for eye dominance effects (cf. Fig. 3B), we also included the categorical variable eye of origin (of the matching grating) into the model, which coded whether the stimulus that matched the predictive context was presented to the nondominant or the dominant eye. In addition, because the imbalance in eye dominance was graded in both the children and adult participants (cf. Fig. 2), and our goal was to control for this additional source of individual differences, we also included eye imbalance as a continuous predictor variable. Eye imbalance was computed from the ratio of total dominance durations for the dominant vs. the dominant eye from the consecutive rivalry experiment. Based on this ratio, we rank-ordered participants from more balanced (participants with very similar percept durations for the dominant and the nondominant eye) to more imbalanced (participants with much higher percept durations for the dominant compared to the nondominant eye). Within each group, the continuous predictor variables (eye imbalance, RME, SST ToM, and SST Physical) were Z-scaled, so that participants with lower values were assigned negative Z-scores and participants with higher values were assigned positive Z-scores.

**Figure 5:**
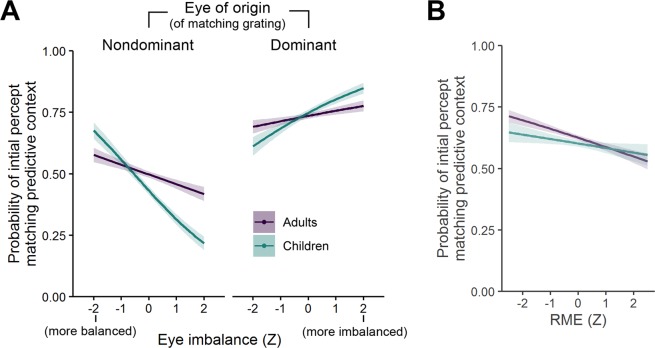
Conditional probability of the initial percept matching the predictive context, based on a GLMM analysis of single-trial data. Lines and shaded areas represent fitted values and standard errors using the R package ‘effects^62^’. (**A**) Significant interaction effect (P < 0.001) of Group × Eye of Origin × Eye Imbalance. (**B**) Significant main effect of RME (P < 0.001), reflecting a slightly negative relationship with the probability that the initial percent matched the predictive context. Note that the interaction effect of Group × RME was not statistically significant. For detailed statistics, see Table 2.

The fixed effects from the GLMM analysis are listed in Table 2. The analysis yielded a significant interaction of Group × Eye of Origin × Eye Imbalance (P < 0.001), which confirmed that the probability of initially perceiving the stimulus that matched the predictive context was higher when this stimulus was presented to the dominant eye. Moreover, this effect was qualified by the eye imbalance of the individual participants, such that participants who had more balanced dominance durations (in the consecutive rivalry experiment) also showed a smaller difference between the dominant and nondominant eye with regard to the matching effect in the predictive context experiment. Accordingly, participants who showed a steep imbalance between the dominant and the nondominant eye in the consecutive rivalry experiment showed a much higher asymmetry in the matching effect between the dominant and the nondominant eye. Furthermore, this moderating influence of eye imbalance was more pronounced in children compared to adults (see Fig. 5A).

**Table 2:** Fixed effects estimates from a GLMM with the binary outcome variable of the initial percept matching the predictive context from single trial data (1 = Initial Percept Match; 0 = Initial Percept Mismatch). Reference levels for contrasts: Adults/Nondominant eye. Effects ordered according to z value from largest to smallest. Model formula: Initial Percept Match ~ Group * Eye of Origin * Eye Imbalance + Group * RME + Group * SST ToM + Group * SST Physical + (1 | Participant). Continuous predictor variables were Z-scaled within groups.

Modeled fixed effects	*β*	SE	z	P
(Intercept)	−0.0106	0.0527	−0.20	0.841
*Significant effects*				
Eye of Origin	1.0357	0.0655	15.81	< 0.001
Group × Eye of Origin × Eye Imbalance	0.5510	0.1059	5.20	< 0.001
Eye of Origin × Eye Imbalance	0.2699	0.0656	4.12	< 0.001
Group × Eye Imbalance	−0.3451	0.0885	−3.90	< 0.001
RME	−0.1589	0.0480	−3.31	< 0.001
Group × Eye of Origin	0.3293	0.1052	3.13	0.002
Group	−0.2644	0.0848	−3.12	0.002
Eye Imbalance	−0.1602	0.0534	−3.00	0.003
*Nonsignificant effects*				
Group × SST Physical	−0.1331	0.0896	−1.48	0.138
Group × SST ToM	0.1310	0.0931	1.41	0.159
SST Physical	0.0643	0.0552	1.17	0.244
Group × RME	0.0818	0.0820	1.00	0.318
SST ToM	−0.0466	0.0549	−0.85	0.397

From the ToM measures, only the RME scores had a significant effect on the probability that the initially perceived percept matched the predictive context (see Fig. 5B). However, the individual RME scores and the probability of the initial percept matching the predictive context were negatively associated, which means that, contrasting the initial hypothesis, participants who achieved higher scores in the RME test tended to show smaller predictive context effects with regard to BR onset, and vice versa, even after controlling for the effects of sensory eye dominance. Despite the generally higher performance of adults in the RME test, the relative differences between individuals did not interact with the age group. In addition to the full GLMM analysis of single trial data, we also fitted a simpler linear fixed effects model with the continuous outcome variable of % trials in which the initial percept matched the predictive context (across dominant and nondominant eye). The results confirmed the significant negative relationship between RME scores and predictive context effects, *β* = −2.79 (SE = 1.18), t =  −2.50, P = 0.014 (all other predictor effects with P ≥ 0.279, see Table S1).

## Discussion

Predictive coding models suggest that prior experience and knowledge shape early perceptual processing^3,22^ as well as social cognitive skills such as ToM^27,28^. Here, we studied predictive effects on early visual perception in children and adults using BR and tested their potential relationship to individual variability in tests of ToM. In both age groups, a predictive context had a similar substantial influence on perception at the onset of BR, suggesting that predictive effects at early perceptual stages are already fully developed in 12-to-13-year-old children. In contrast, we observed substantial developmental effects for the RME test as well as numerically higher performance for adults compared to children in the SST, in line with previous reports of ToM-related improvements from childhood to adulthood^31^. For assessing the relationship between predictive effects at early perceptual stages and individual performance in ToM, we also controlled for eye dominance, as a potentially important additional source of variability for BR^13,48,49^. This joint analysis revealed that participants’ eye imbalance (the degree to which one eye is more dominant than the other) explained a substantial amount of individual differences in BR onset effects. However, we did not observe the hypothesized positive relationship between predictive context effects and ToM measures. In opposition to our initial hypothesis, individual performance in the RME test was negatively associated with the predictive context effect on BR. For the SST measures, we found no association whatsoever with individual variability in predictive effects on BR. Thus, the obtained data argues against our initial hypothesis of a positive relationship between predictive effects on early perceptual processing and higher-level ToM-related skills.

As our initial hypothesis was directional, any interpretation of the negative relation between the RME test and BR is consequently purely speculative and needs to be followed up in further research. For example, one possible explanation for this pattern of results might be that a focus on low-level visual aspects (e.g., orientation and apparent motion direction) is required for participants to integrate contextual information in the BR task. In contrast, a focus on the semantic interpretation of the visual images is required for the RME test. If participants are more inclined to focus on low-level visual features, they may be less able to focus on the semantic interpretation and vice versa. As the SST does not use visual images but rather verbal stories, the negative relation does not occur with this test. The finding that only the RME but not the SST correlated negatively with BR context effects supports the view that these two commonly used measures of the ToM construct tap into different aspects of social cognition^50^. It is also noteworthy that the current and previous research with orientation stimuli^22,23^ consistently found that expected stimuli were preferably perceived at BR onset. In contrast, the opposite pattern of results was reported for more complex photographic stimuli of different semantic categories, namely a preference for unexpected stimuli^24^. Furthermore, it is possible that presenting discrete orientations of sinusoidal gratings measures the degree to which an object is holistically integrated over time, whereas this specific aspect of perceptual processing is not required for correctly solving the RME test. Thus, further research is necessary to understand whether the particular choice of stimuli and test materials determines the reliability and direction of the association between BR and ToM measures.

Given the lack of reliable implicit ToM measures^40^, the present study can be regarded as a starting point to explore the use of established experimental paradigms for assessing developmental changes in potentially critical ToM-related perceptual effects. We reasoned that predictive context effects on BR could inform on the individual disposition for including prior knowledge into one’s own perceptual experience, which might also be critical for understanding the inner states and beliefs of others and predicting their actions. The fact that, despite the considerable individual variability, BR prediction effects were not positively associated with ToM could mean that predictive mechanisms operate at many different neural sites and can influence perceptual and cognitive processing in various ways^51^. Thus, the influence of prior knowledge on ToM-related skills might stem from different neurocognitive mechanisms than the predictive effects operating at early perceptual stages, which were probed by our present BR experiments. Numerous different abilities might be involved in ToM-related social cognition, including emotion recognition, social attention, biological motion perception, and others^52^, with the reliable integration of predictive, contextual information being only one of these many aspects. Moreover, in line with our current results, different developmental trajectories could be associated with predictive effects involved in BR and ToM, respectively.

The age-related improvements on ToM scales suggest that these abilities, as opposed to BR effects, might undergo further development from childhood to adulthood, possibly resulting from brain maturation in the frontal cortex^53,54^. ToM measures correlated with another in both adults and children, suggesting that they measure similar underlying concepts. This is in line with a number of studies showing correlations between specific ToM tasks^55–57^. Moreover, the correlation between ToM and physical scales of the SST may suggest that the mechanisms underlying both measures reflect some higher-level cognitive construct instead of skills that are exclusive to ToM. In agreement with this interpretation, the developmental changes may reveal themselves more clearly in the RME, which measures perception-related aspects of ToM more directly, as potential confounds with general cognitive capacities might be avoided. This is in line with the current debate questioning whether implicit ToM measures might also reflect more parsimonious mechanisms rather than mindreading^40,58–60^.

We did not find any overall age differences in BR dynamics, neither concerning consecutive rivalry, where such differences were previously reported for younger children^15–17^, nor concerning predictive effects on BR onset, which was not studied in children so far. Note that some previous studies tested younger children up to 6 years^17^. While Hudak *et al*.^16^ reported a developmental trend from 9-year olds to 21-year olds with more extensive average dominance durations in adults compared to children, the 12-year old children included in their study did not show statistically robust differences from the other two age groups, when compared directly. It is important to point out that these previous developmental studies did not look at predictive context effects and their relationship with individual ToM skills, which were novel research questions in our current study. Nevertheless, it could be that in our sample of 12 to 13 year-old participants, possible developmental changes in BR context effects may already have been completed, explaining the similar overall pattern of results in adults and children. One difference between children and adults, which we observed in our study, though, concerns the extent to which the imbalance between the dominant and the nondominant eye modulated the predictive effects on BR onset. Specifically, the eye imbalance caused a steeper modulation in the predictive effect on BR onset in children compared to adults (cf. Fig. 5A). One possible interpretation could be that sensory eye dominance plays a more substantial role in children, possibly pointing to a developmental shift from the low-level influence of eye dominance in children towards a stronger impact of top-down signals attenuating the eye dominance effects in adults. However, because we did not predict this effect, this interpretation is only speculative. It needs to await confirmatory testing in an independent sample of participants before theoretical implications could be formulated.

From a methodological point of view, the current study makes two contributions. First, the paradigm for studying predictive context effects on BR onset has proven well-suited for investigations in 12-to-13-year-old children, with no obvious procedural shortcomings compared to conducting the same experiments in adults. Hence, the next step will be to extend these investigations to younger age groups and determine the developmental stage at which the predictive effects start to emerge. Given that the perceptual reports collected in the present BR paradigm do not reflect objective performance, and also do not directly benefit from repeated testing, the paradigm would also be suited for longitudinal studies of perceptual development within the same child participants. Age-related changes in predictive context effects could reflect the development of top-down influences in basic perceptual processes without the potential confound of general knowledge, which would not be possible, for example, with explicit ToM tests. The second methodological contribution of the current study is the use of a head-mounted display to investigate binocular rivalry. Compared to other solutions such as mirror stereoscopes^61^, which require participants to sit tight and place their head in a chin rest, the HMD allows participants to move more naturally during the experimental task while ensuring that the correct input is viewed on each eye. It is, therefore, more comfortable for participants and well suited for testing children.

### Conclusion

The present study tested to which extent a predictive context biases visual experience in BR in children and adults, and whether individual differences in perceptual effects are related to abilities to understand socially relevant information, as measured by two established ToM scales. We extend prior results by showing that predictive context effects on early perceptual processing are fully developed in 12 to 13-year-old children while social cognition still improves from childhood until adulthood. In children and adults, predictive effects on BR were not positively associated with performance in explicit ToM scales. Instead, individual differences in BR onset effects were explained mainly by differences in sensory eye dominance.

## Supplementary information

Supplementary Information.

## Data Availability

Primary data and R code to reproduce the analyses are available through the Open Science Framework (https://osf.io/cx8r9/).
