# Supplementary material for: Predictive context biases binocular rivalry in children and adults with no positive relation to two measures of social cognition
Source: Sci Rep. 2020 Feb 6;10:2059. doi: 10.1038/s41598-020-58921-8 (PMC7005192; doi:10.1038/s41598-020-58921-8)
Supplement: Supplementary file 1 — Supplementary Information. [file 41598_2020_58921_MOESM1_ESM.pdf]

Supplementary Information for the manuscript:

Valuch, C. & Kulke, L. (2020). Predictive context biases binocular rivalry in children and adults with no positive relation to two measures of social cognition.

**Modeled effects**	**β**	**SE**	**t**	**P**
(Intercept)	61.81	1.08	57.23	< 0.001
Significant effects				
RME	–2.97	1.19	–2.50	0.014
Nonsignificant effects				
Group	–1.88	1.72	–1.09	0.279
Group × SST ToM	1.98	2.29	0.86	0.390
Eye imbalance	–0.83	1.10	–0.75	0.455
Group × Eye imbalance	–1.27	1.83	–0.69	0.490
Group × RME	1.39	2.01	0.69	0.493
SST ToM	–0.91	1.38	–0.66	0.511
Group × SST Physical	–1.26	2.20	–0.57	0.568
SST Physical	0.72	1.38	0.52	0.604

Table S1. Fixed effects estimates from a linear model with the continuous outcome variable "Initial percept matching predictive context (% trials)". Reference age group: Adults. Effects ordered according to t value from largest to smallest. Model formula: Percent_Matching ~ Group*Eye_Imbalance + Group*RME + Group*SST_ToM + Group*SST_Physical. Continuous predictor variables were Z-scaled within groups.
